# Overcoming Chemoresistance via Extracellular Vesicle Inhibition

**DOI:** 10.3389/fmolb.2021.629874

**Published:** 2021-03-24

**Authors:** Raeesah Hayatudin, Zhijack Fong, Long Chiau Ming, Bey-Hing Goh, Wai-Leng Lee, Nurolaini Kifli

**Affiliations:** ^1^School of Science, Monash University Malaysia, Subang Jaya, Malaysia; ^2^PAP Rashidah Sa’adatul Bolkiah Institute of Health Sciences, Universiti Brunei Darussalam, Gadong, Brunei; ^3^College of Pharmaceutical Sciences, Zhejiang University, Hangzhou, China; ^4^Biofunctional Molecule Exploratory (BMEX) Research Group, School of Pharmacy, Monash University Malaysia, Subang Jaya, Malaysia

**Keywords:** chemoresistance, extracellular vesicle, cancer, exosomes, cell death

## Abstract

With the ever-growing number of cancer deaths worldwide, researchers have been working hard to identify the key reasons behind the failure of cancer therapies so the efficacy of those therapies may be improved. Based on extensive research activities and observations done by researchers, chemoresistance has been identified as a major contributor to the drastic number of deaths among cancer patients. Several factors have been linked to formation of chemoresistance, such as chemotherapy drug efflux, immunosuppression, and epithelial-mesenchymal transition (EMT). Lately, increasing evidence has shed light on the role of extracellular vesicles (EVs) in the regulation of chemoresistance. However, there is limited research into the possibility that inhibiting EV release or uptake in cancer cells may curb chemoresistance, allowing chemotherapy drugs to target cancer cells without restriction. Prominent inhibitors of EV uptake and release in cancer cells have been compiled and contrasted in this review. This is in the hope of sparking greater interest in the field of EV-mediated chemoresistance, as well as to provide an overview of the field for fundamental and clinical research communities, particularly in the field of cancer resistance research. In-depth studies of EV-mediated chemoresistance and EV inhibitors in cancer cells would spur significant improvement in cancer treatments which are currently available.

## Introduction

Chemoresistance, a state of cancer cells wherein chemotherapy drugs are ineffective against cancer progression, is a major challenge in cancer treatment. It accounts for more than 90% of deaths in patients with metastatic tumors (Wang et al., 2019). Chemoresistance in tumors may be either intrinsic or acquired. Acquired chemoresistance may cause cancer cells to develop insensitivity not only to the particular chemotherapy drug being used, but also to other types of chemotherapy drugs with different modes of action (Wang et al., 2019). Many chemotherapy drugs function by targeting DNA replication, which prevents the growth of cancer cells and induces apoptosis – for example, cisplatin intercalates with double-stranded DNA whereas 5-fluorouracil inhibits DNA nucleotide synthesis (Chorowala et al., 2012). However, chemoresistant cells respond to these drugs by developing mechanisms which promote their proliferation and inhibit apoptotic processes. These mechanisms have recently been proven with the involvement of extracellular vesicles (EVs) (Namee and O’driscoll, 2018; Santos et al., 2018).

This review will compile major drug inhibitors targeting specific mechanisms in the release and uptake of both exosomes and microvesicles (MVs) from cancer cells. Existing literature regarding the effectiveness of these inhibitors, as well as the possible difficulties in using them, will be analyzed. This is with the aim of emphasizing the potential of using EV inhibitors to target chemoresistance in cancer therapies.

### Subtypes of EVs

Extracellular vesicles are small, lipid bilayer-enclosed particles secreted from all types of cells for intercellular communication and the removal of cellular wastes; in cancer cells, they mediate chemoresistance (Fan et al., 2018) as well as cancer progression (Xu et al., 2018). EVs exist in a diverse range of sizes even within subclassifications. Their constituents (a mix of nucleic acids, proteins and lipids) may vary depending on the subtype of EV, the function of the parent cell (Zaborowski et al., 2015), the stage of cell growth or pathogenesis the parent cell is undergoing (Becker et al., 2016), and even whether EVs are secreted from the apical or basolateral surfaces of polarized cells (Tauro et al., 2013; Chen et al., 2016). The two most well-categorized EV subtypes concerning chemoresistance are exosomes and MVs. Exosomes are typically defined as EVs falling within the size range of 30 to 100 nm, resulting directly from endosomal multivesicular bodies (MVBs); whereas MVs are classified as being within 100 to 1000 nm and outwardly budding from cell membranes (Doyle and Wang, 2019; Ciardiello et al., 2020). Nevertheless, EVs in the size range of exosomes have been found to occasionally derive from outward budding rather than endosomal MVBs, though not yet in cancer cell lines (Lenassi et al., 2010; Abels and Breakefield, 2016).

### Biogenesis and Release of EVs

Exosomes are formed via the fusion of MVBs with the plasma membrane; MVBs result from the inward budding of endosomal membranes (Huotari and Helenius, 2011). MVBs are assembled via endosomal sorting complexes required for transport (ESCRT)-independent and ESCRT-dependent pathways (Huotari and Helenius, 2011). In the ESCRT-independent pathway, sphingomyelinase enzymes (SMases) convert sphingomyelin (present in endosomal membranes within lipid rafts) to ceramide (Catalano and O’driscoll, 2020). The ceramides which associate to form microdomains which drive the formation of MVBs from intra-luminal vesicles (ILVs) (Huotari and Helenius, 2011). Meanwhile, the ESCRT-dependent pathway requires ESCRT protein complexes (specifically, ESCRT-0, ESCRT-I, ESCRT-II, and ESCRT-III) with associated proteins (such as VPS4, VTA1, and ALIX) which aid in the generation of ILVs (Huotari and Helenius, 2011). ESCRT-0 gathers ubiquitinated proteins and recruits ESCRT-I and ESCRT-II, which cooperate to contort the endosomal membrane and sort the constituents of the forming ILV (Hanson and Cashikar, 2012; Taylor and Bebawy, 2019). ESCRT-III component proteins are gathered by ESCRT-II or ALIX, and polymerization of those components causes neck constriction and cleavage to form ILVs (Catalano and O’driscoll, 2020). Vsp4, an ATPase, disassembles the ESCRT and allows them to be recycled (Babst, 2011). Farnesyltransferase enzymes are necessary for the activation of Ras proteins, which, together with its downstream effectors (including Raf and extracellular signal-regulated kinases, ERK), have been implicated in exosome biogenesis (Sexton et al., 2019). The MVBs formed which do not fuse with lysosomes are transported toward the cell membrane with the aid of cytoskeletal actin and microtubules; this process is regulated by several proteins such as the Rab proteins (Bobrie et al., 2012), and cholesterol impacts it as well (Pfrieger and Vitale, 2018). It should be noted that the ESCRT-independent and ESCRT-dependent pathways might actually coordinate together in the biogenesis of exosomes rather than function distinctly (Babst, 2011; Hessvik and Llorente, 2018). Genetic editing to knock out the components of ESCRT-independent and ESCRT-dependent mechanisms would help to clarify whether those components are truly necessary for exosome biogenesis, and may reveal the extent to which the mechanisms cooperate (Maas et al., 2017).

Microvesicles form via budding directly from the plasma membrane. The transport of molecular constituents of MVs toward the plasma membrane as well as the rearrangement of membrane lipids and actin cytoskeletal components are integral to this process (Hessvik and Llorente, 2018). Several unique mechanisms involving lipids relate to MV release, including the externalization of phosphatidylserine (PS) at specific locations of the cell membrane where microvesiculation occur (Tricarico et al., 2017), as well as acid sphingomyelinase (aSMase)-catalyzed conversion of sphingomyelin to ceramide, which induces the curving of the plasma membrane and subsequent MV release (Muralidharan-Chari et al., 2010; Awojoodu et al., 2014; Hoehn et al., 2017; Menck et al., 2017). Cholesterol is involved in MV release in addition to intracellular transport of MVBs, although its precise role is unknown (Pfrieger and Vitale, 2018). Cytoskeleton-interacting components and regulators of those components also participate in the biogenesis of MVs. These include the Rho family of GTPases, which takes part in many cell signaling processes such as cytoskeleton reorganization (Catalano and O’driscoll, 2020), most prominently among them, RhoA, which acts on Rho-associated protein kinases (ROCK) and ERK (Li et al., 2012; Sedgwick et al., 2015; Hoehn et al., 2017). ROCK acts on the cytoskeleton to influence the cellular shape and movement (Hartmann et al., 2015). RhoA acts through the RhoA-cofilin pathway to regulate the rearrangement of cytoskeletal actin in microvesiculation (Wilson et al., 2013). RhoA along with ADP-ribosylation factors 6 and 1 (ARF6 and ARF1) additionally increase myosin contractility via phosphorylation of myosin light chains, hence encouraging fission and the release of MVs (Schlienger et al., 2014). In addition, ESCRT proteins directly participate in MV biogenesis by regulating the cleavage of the plasma membrane to release MVs (Nabhan et al., 2012; Hurley, 2015; Wang and Lu, 2017; Arii et al., 2018; Taylor and Bebawy, 2019). In this way, MV biogenesis parallels the formation of ILVs, nonetheless, while the involvement of ESCRT in ILV formation is clear, more investigation is required regarding the role of ESCRT in MV budding. Moreover, this suggests that the distinction between the pathways of exosome biogenesis versus those of microvesiculation may not always be so clear-cut. More research is needed in order to elucidate the mechanisms of microvesiculation.

### Uptake of EVs

Past research (Tian et al., 2014; Costa Verdera et al., 2017) has shown that EVs (including exosomes and MVs) are endocytosed by cancer cells through two main routes of pinocytosis, which can be broadly classified as clathrin-dependent endocytosis (CDE) and clathrin-independent endocytosis (CIE). In CDE, major coat proteins and endocytic accessory proteins assemble to form clathrin-coated pits and the GTPase dynamin assists in the scission of endocytic vesicles (Mettlen et al., 2018). In comparison, CIE encompasses a number of pathways including macropinocytosis as well as dynamin-independent processes such as CDC42- and ARF6-dependent endocytosis, and dynamin-dependent processes such as caveolae- and RhoA-dependent endocytosis (Dutta and Donaldson, 2012; Iversen et al., 2012; Costa Verdera et al., 2017). Many of the aforementioned variations of CIE are cholesterol-dependent (Dutta and Donaldson, 2012). Several modes of macropinocytosis exist which may be dynamin-dependent and dynamin-independent (Hetzenecker et al., 2016; Sandvig et al., 2018), although further research is needed to clarify these mechanisms. Investigations into whether CDE or CIE are more significant in the uptake of EVs by cancer cells have yielded conflicting results, depending on the types of cell lines used. For example, in mesenchymal cells taking up PC12 (rat pheochromocytoma)-derived exosomes, CDE, and macropinocytosis (but not any other pathways of CIE) were implicated in uptake processes (Tian et al., 2014). However, for Jeko-1 and Mino (mantle cell lymphoma) cells (Hazan-Halevy et al., 2015), as well as A431 (human epidermoid carcinoma) and HeLa cells (Costa Verdera et al., 2017), EVs were described broadly to be internalized via CIE, but not CDE. EV uptake mechanisms in cancer cells may depend on cell types which produce and accept EVs.

### Regulation of Chemoresistance by EVs

**FIGURE 1:**
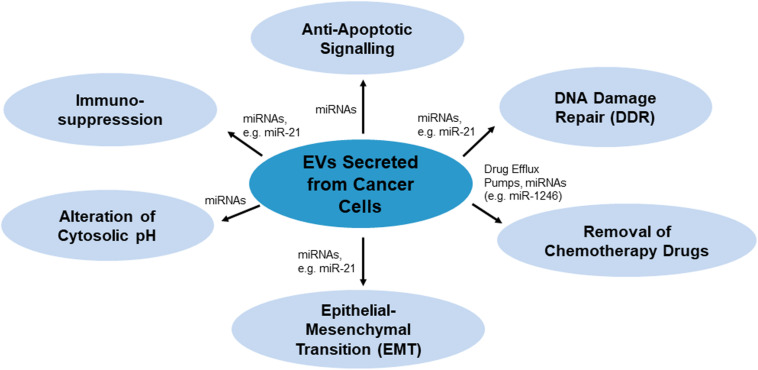
Mechanisms of EVs-mediated chemoresistance miRNAs or proteins such as drug efflux pumps as the key players carrying drug resistance messages.

There are various mechanisms by which EVs confer chemoresistance from chemoresistant cancer cells to chemosensitive cells. These mechanisms have been summarized in Figure 1. As per Figure 1, EVs may modulate chemoresistance by transferring vesicular content, especially microRNAs (miRNAs), which activate anti-apoptotic signaling and DNA damage repair (DDR) (miR-21), and by enhancing other processes such as chemotherapy drug efflux (miR-1246), immunosuppression (miR-21), alteration of cytosolic pH and epithelial-mesenchymal transition (EMT) (miR-21) (Cereghetti and Lee, 2014; Yousafzai et al., 2018; Maacha et al., 2019; O’neill et al., 2019).

MicroRNAs, small single-stranded non-coding RNAs about 19–25 nucleotides long, contribute to the development of chemoresistance by influencing genes relating to drug resistance, cell survival and proliferation, cell cycle, apoptosis, stress tolerance and immunity (Si et al., 2019). MiRNAs regulate various genes via inhibiting mRNA translation. In EV-mediated chemoresistance, many studies suggested miRNA play important roles in the process. For instance, paclitaxel resistant ovarian cancer cells were reported to secrete EVs with abundance of miR-1246. Transfer of this miRNA promotes drug resistance phenotype in recipient cells by inhibiting the expression of Cav1 and upregulating ABCB1 expression to facilitate paclitaxel efflux (Kanlikilicer et al., 2018). Gemcitabine is a DNA chelator that is activated by deoxycytidine kinase. Several miRNAs were found to regulate the transferring of gemcitabine resistance. miR-365 in EVs secreted by tumor associated macrophages was observed to induce resistance of pancreatic ductal adenocarcinoma cells in gemcitabine treatment. This miRNA increased the concentration of triphosphate nucleotides (NTPs) in the recipient cells to allow more NTPs competing with activated gemcitabine, which effectively decreased the efficacy of gemcitabine (Binenbaum et al., 2018). On the other hand, increased miR-106 was found in EVs secreted by cancer associated fibroblasts when exposed to gemcitabine. Uptake of miR-106 enriched EVs contributes to the resistance of pancreatic cancer cells (AsPC-1) against gemcitabine treatment. miR-106 conferred resistance toward gemcitabine by binding onto its target gene, TP53INP1, suppressing the expression of the protein (Fang et al., 2019).

Extracellular vesicles may additionally contribute to producing a chemoresistive tumor microenvironment (Maacha et al., 2019). miRNA in EVs could alter chemotherapy response and promote tumorigenesis by mediating the intracellular communication between the tumor and healthy cells. For examples, the tumor-derived EVs in patients with hepatocellular carcinoma (HEPG2) was found to contain miRNA-1247-30, a miRNA facilitates metastatic invasion of the tumor. It promotes the differentiation of normal lung fibroblasts into tumor promoting cancer-associated cells. The cancer-associated fibroblasts further enhance expression of pro-inflammatory cytokines such as interleukin-6 and interleukin-8 in cancer cells that led to their resistance in sorafenib treatment (Fang et al., 2018). Together these findings implicate inhibition of EVs may solve the problem of insensitivity of cancer cells in response to clinical drugs like gemcitabine and sorafenib.

### Inhibition of EVs

In investigations into EV-mediated chemoresistance, treatment of cancer cell lines with EV-inhibiting drugs may serve two functions: to confirm that EVs contribute to chemoresistance, or to test whether EV inhibition is a suitable strategy to complement treatment with chemotherapy drugs. Although clear links have been established regarding the contribution of EVs to chemoresistance, scientific understanding of the precise mechanisms associated remain primitive and requires more investigation. EV inhibitors which bind to certain proteins or deregulate genes implicated in inducing chemoresistance may be used to clarify the roles those proteins or genes play in chemoresistance. An EV-related strategy against chemoresistance which holds promise includes inhibiting the biogenesis, release or uptake of EVs by cancer cells. Another approach of extracting tumor-derived EVs out from the tumor microenvironment using specific markers for those EVs has also been proposed (Maacha et al., 2019), but this method has not been investigated for its efficacy using cancer cell lines at this time. While the inhibition of EV biogenesis, release or uptake may involve unique challenges due to the lack of specificity of some inhibitor drugs, it has proven efficient in inducing chemosensitivity to previously chemoresistant cancer cells. For instance, the kinase inhibitor U0126 sensitized chemoresistant Suit-2 (human pancreatic adenocarcinoma) cells to the chemotherapy drug gemcitabine, although it did not have as strong an effect on another chemoresistant pancreatic cancer cell line (Muralidharan-Chari et al., 2016). In the future, EV inhibition may prove to be an effective clinical strategy to counteract chemoresistance; for this purpose, the targeted pathways of EV production and uptake of EV-inhibiting drugs must be further understood.

To encourage further investigation, 33 inhibitors have been assessed according to the pathways of EV secretion or uptake which they primarily target. These pathways, as well as their corresponding inhibitors, have been summarized in Table 1, according to current knowledge about the activities of the inhibitors. Nevertheless, EV inhibitors may target more than one pathway, either due to their unspecificity or because the subject of the inhibitor may participate in multiple pathways of EV release and uptake. The inhibitors may target exosomes or MVs, or both.

**TABLE 1:** List of EV inhibitors.

Targets of inhibition	Reported inhibitors	Cell line or model used	References
Shedding of MVs	Bisindolylmaleimide-I	Cell lines: PC3, MCF-7	Kosgodage et al., 2017
	Calpeptin	Cell line: PC3	Jorfi et al., 2015
	d-pantethine	Cell line: PC3	Kosgodage et al., 2017
	Glyburide	Cell lines: PC3 and MCF-7	Kosgodage et al., 2017
	NSC23766	Sepsis model using mice	Wang et al., 2017
	U0126	Cell lines: Suit-2, MPanc-96	Becker et al., 2016
	Y27632	Cell lines: PC3 and MCF-7	Kosgodage et al., 2017
Formation of exosomes	Cambinol*	Cell line: NCI-H460	Heltweg et al., 2006
	Cytochalasin D	Cell lines: HeLa, Panc 1, PC3, and A293	Khan et al., 2011
	Dasatinib	Cell line: K562	Mineo et al., 2012
	Dimethyl amiloride (DMA)	Cell lines: CT26, EL4, and H23	Chalmin et al., 2010
		Model: three mouse tumor models using two mouse cancer cell lines, EL4 and TS/A, and one human cancer cell line, CT26	
	GW4869	Cell line: SW620 cancer stem cells	Hu et al., 2015
	Imatinib	Cell line: K562	Mineo et al., 2012
	Indomethacin	Cell lines: SU-DHL-4, OCl-Ly1 and OCl-Ly3	Koch et al., 2016
	Ketotifen	Cell lines: HeLa, MCF-7 and BT549	Khan et al., 2018
	Manumycin A	Cell lines: C4-2B, PC3, and 22Rv1	Datta et al., 2017
	MβCD	Cell lines: PC3 and MCF-7	Kosgodage et al., 2017
	Simvastatin	Non-cancerous cell lines: Beas-2B and THP-1	Kulshreshtha et al., 2019
	Spiroepoxide*	Cell line: PC3	Phuyal et al., 2014
	Sulphisoxazole	Cell lines: MCF-7, MCF-10A, and MDA-MB-231	Im et al., 2019
	Tipifarnib	Cell line: modified C4-2B expressing exosomal marker CD63	Datta et al., 2018
Formation of both MVs and exosomes	Cannabidiol	Cell lines: HEPG2, MDA-MB-231, and PC3 cell lines	Kosgodage et al., 2018
	Chloramidine (Cl-amidine)	Cell lines: PC3 and MCF-7	Kosgodage et al., 2017
	Imipramine	Cell lines: PC3 and MCF-7	Kosgodage et al., 2017
	SMR peptides	Cell lines: MCF-7 and MDA-MB-231	Huang et al., 2019
Clathrin-dependent endocytosis (CDE)	Chlorpromazine	Non-cancerous cell lines: HuH-7, Vero, COS-7, ARPE-19, and D407	Vercauteren et al., 2010
	Dynasore	Non-cancerous cell lines: HuH-7, Vero, COS-7, ARPE-19, and D407	Dutta and Donaldson, 2012
	Ikarugamycin (IKA)	Cell lines: H1299, HCC366, and H1437	Elkin et al., 2016
	MβCD	Cell lines: PC3 and MCF-7	Kosgodage et al., 2017
Clathrin-independent endocytosis (CIE)	Genistein	Cell lines: A2780, CaOV3, ES2, and SK-OV-3	Costa Verdera et al., 2017
	Heparin	Cell line: U87	Christianson et al., 2013
	MβCD	Cell lines: PC3 and MCF-7	Kosgodage et al., 2017
	Simvastatin	Non-cancerous cell lines: Beas-2B and THP-1	Kulshreshtha et al., 2019
Macropinocytosis (subset of CIE)	Cytochalasin D	Cell lines: A431 and HeLa	Costa Verdera et al., 2017
	Dimethyl amiloride (DMA)	Cell lines: CT26, EL4 and H23	Chalmin et al., 2010
		Model: three mouse tumor models using two mouse cancer cell lines, EL4 and TS/A, and one human cancer cell line, CT26	
	EIPA	Cell line: 4T1	Lin et al., 2018
	NSC23766	Cell lines: MDA-MB-435 and MCF-10A	Hernandez et al., 2010
Both CDE and CIE	Chloramidine (Cl-amidine)	Non-cancerous cell lines: HuH-7, Vero, COS-7, ARPE-19, and D407	Vercauteren et al., 2010

*Inhibitors which have not yet been tested with cancer cells (categorization based on mechanism of inhibitor drug).

The 33 inhibitors discussed may be broadly categorized as inhibitors of lipid-related pathways, cytoskeletal organization, other miscellaneous pathways of EV release and endocytosis. These four classifications have been selected based on the specific mechanisms targeted by each inhibitor.

### Inhibitors of Lipid-Related Pathways

**FIGURE 2:**
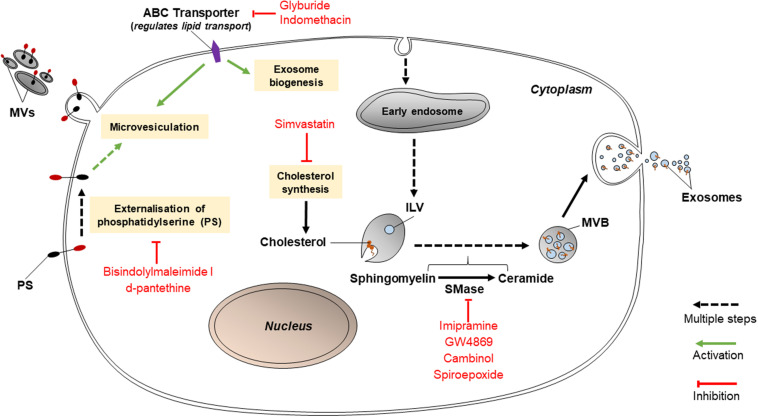
Inhibitors of lipid-related pathways (bisindolylmaleimide-I, d-pantethine, glyburide, indomethacin, simvastatin, imipramine, GW4869, cambinol, and spiroepoxide) inhibit EV biogenesis.

Various lipid-related mechanisms are associated with the release of EVs from cancer cells. These mechanisms primarily involve the conversion of sphingomyelin to ceramide by sphingomyelinases, PS translocation by protein kinases, and cholesterol synthesis. Ceramides play roles in causing the curvature of membranes prior to the formation of MVBs or release of MVs; PS translocation is a necessary step for MVs to be released; and cholesterol influences the formation of MVBs and MVs (Figure 2). Thus, inhibition of any of these pathways may hold promise in reducing EV release. If so, this may disable cancer cells from transmitting chemoresistance to other cancer cells.

#### Inhibitors Targeting Sphingomyelinases

Such inhibitors include imipramine, GW4869, cambinol, and spiroepoxide as depicted in Figure 2. GW4869, cambinol, and spiroepoxide selectively inhibit neutral sphingomyelinases (nSMase), more specifically neutral sphingomyelinase 2 (nSMase2); whereas imipramine targets aSMase. Each inhibitor may have a different specific mechanism, but they have all been proven to reduce exosome secretion, to varying levels of efficiency. For example, imipramine, an aSMase inhibitor, was found to block the release of exosomes and MVs from glial cells (Bianco et al., 2009). aSMases catalyze the hydrolysis of sphingomyelin to ceramide, which is vital to the release of exosomes and the formation of MVs. When MVs are shed, aSMases are translocated to the cell membrane; once internalized in cells, imipramine acts to proteolytically degrade aSMase. aSMase then detaches from the cell membrane (Catalano and O’driscoll, 2020). A study by Kosgodage et al. (2017) proved that 25 μM imipramine lessened total EV release (both exosomes and MVs) by 77% in PC3 (prostate cancer) cells although the separate effects of imipramine on exosomes and on MVs was not tested (Kosgodage et al., 2017). Imipramine may also be used to inhibit macropinocytosis.

nSMase2 inhibitors block ceramide-mediated exosome biogenesis. GW4869 is a frequently used inhibitor which targets nSMase2 to prevent the ceramide-modulated inward budding of MVBs and the subsequent release of exosomes from MVBs (Catalano and O’driscoll, 2020). GW4869 was used to suppress chemoresistance in colorectal, pancreatic and ovarian cancer cells by reducing exosome secretion (Hu et al., 2015; Cao et al., 2017; Richards et al., 2017). Cambinol, a lipid molecule, binds to the active site of nSMase2 (Figuera-Losada et al., 2015). While its specific nSMase2 inhibitory effect on cancer-derived EVs has not been studied, on the basis of its additional inhibition of NAD-dependent deacetylase activity of cell survival enzymes, it has been found to have antitumorigenic effects on lung cancer cells from the cell line NCI-H460 as well as sensitize those cells to chemotherapy drugs such as DNA-damaging etoposide and tubulin-targeting paclitaxel (Heltweg et al., 2006). Spiroepoxide specifically and non-competitively inhibits nSMase2 (Phuyal et al., 2014) although its ability to diminish exosome secretion in cancer cells has not been documented. GW4869 and spiroepoxide have both been found to be non-toxic to cells (Phuyal et al., 2014). It must be noted that while nSMase2 inhibitors have been proven to reduce exosome release in cancerous cell lines as well as some non-cancerous cell types such as HEK293 cells, a human kidney cell line (Kosaka et al., 2010), they do not affect exosome secretion at all in other cell types such as PC3, a prostate adenocarcinoma cell line (Phuyal et al., 2014). The efficiency of EV inhibitors varies between different cell lines and cell types.

#### Inhibitors Targeting PS Translocation

These include bisindolylmaleimide-I and d-pantethine. Bisindolylmaleimide-I, an inhibitor targeting the ATP-binding site of various protein kinase C isoforms, hinders the release of calcium and the externalization of PS, which are known steps in the mechanism driving the release of MVs (Kosgodage et al., 2017). Stratton et al. (2015) detailed that bisindolylmaleimide-I inhibited MV release in PC3 cells by 75% compared to the control, despite intracellular calcium concentrations being increased using the sublytic membrane attack complex (MAC) (Stratton et al., 2015). Meanwhile, d-pantethine, a derivative of vitamin B5, is needed in the production of coenzyme A. It strongly reduces the total levels of cholesterol in cells via its lipid-metabolizing activity (Kosgodage et al., 2017). It also diminishes the shedding of MVs (Kosgodage et al., 2017). Similarly to bisindolylmaleimide-I, it inhibits the translocation of PS (Martin et al., 2016) and therefore prevents microvesiculation. However, d-pantethine severely reduces cell viability by nearly 80% in PC3 cells (Kosgodage et al., 2017).

#### Inhibitors Targeting ATP-Binding Cassette Transporters or Other Proteins Within Cell

Inhibitors targeting ATP-binding cassette (ABC) transporters include glyburide (glibenclamide) and indomethacin. Glyburide, a drug for diabetes, inhibits the ATP-sensitive K^+^ channel of an ABC transporter participating in the release of MVs (Catalano and O’driscoll, 2020). Glyburide non-specifically interacts with the SUR receptor (an ABC transporter) and other proteins such as ABCA1, which regulates cellular cholesterol and phospholipid concentrations (Liu and Tang, 2012). Since cholesterol is vital to the release of both MVs and exosomes, glyburide should be able to have both exosome-inhibiting and MV-inhibiting abilities. Even so, Kosgodage et al. (2017) showed that glyburide did not affect MV biogenesis in PC3 and MCF-7 cells (Kosgodage et al., 2017) and its effect on exosomes was not tested in the study. In comparison, indomethacin, an anti-inflammatory drug, specifically downregulates the transcription of ABCA3, which aids in the transport of lipids (Aung et al., 2011). This explains why indomethacin was able to act as a chemosensitizer for lymphoma cell lines SU-DHL-4, OCl-Ly1 and OCl-Ly3 (Koch et al., 2016). Prior to treatment with indomethacin, the cells encapsulated chemotherapy drugs doxorubicin and pixantrone in exosomes which were then effluxed from cells (Koch et al., 2016). The application of 10 μM indomethacin to the lymphoma cells allowed the drugs to accumulate within their nuclei and exert cytostatic effects (Koch et al., 2016). Although there are many ABC transporter inhibitors such as pelitinib and vatalanib have been identified to sensitize resistant cancer cell lines, e.g., lung and colon cancer cell lines successfully (To et al., 2015a, b), but the study on the effect of these inhibitors in overcoming EV-mediated drug resistance is limited.

Simvastatin, a HMG-CoA reductase inhibitor, prevents the synthesis of cholesterol; since cholesterol is an integral constituent in endosomal membranes, this reduced exosome secretion in epithelial cells and monocytes by 40% (Kulshreshtha et al., 2019). However, simvastatin has not yet been tested with cancerous cell lines. Simvastatin (but not GW4869) was further shown to decrease intracellular concentrations of proteins associated with exosomes such as ALIX, CD63, and CD81 (Kulshreshtha et al., 2019). Simvastatin also inhibits endocytosis (specifically, CIE) dose-dependently (Costa Verdera et al., 2017).

### Inhibitors of Cytoskeletal Organization

**FIGURE 3:**
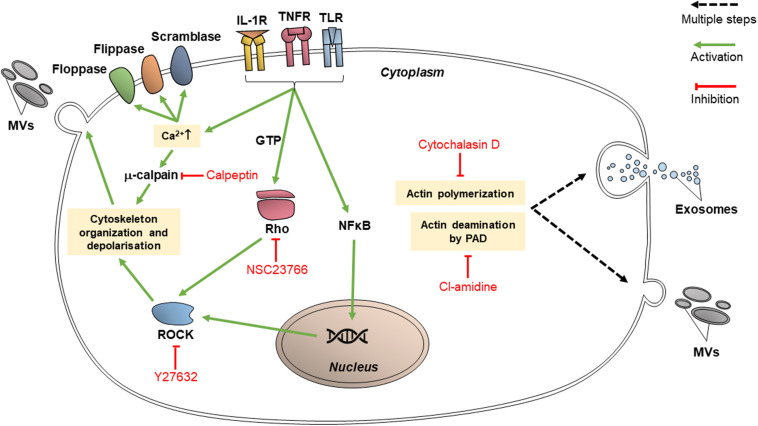
Inhibitors of cytoskeletal organization pathways (calpeptin, Y27632, NSC23766, cytochalasin D, and chloramidine/Cl-amidine) inhibit EV release through intracellular trafficking and budding of the vesicles.

Proteins which participate in cytoskeletal organization are targets for various inhibitors since they are vital for EV release as well as endocytic processes. These proteins may specifically interact with actin; or they may have broad effects on cytoskeleton organization, in which case they are classified as cytoskeleton-related proteins. The targeting of EV release via these pathways may reduce the transfer of vesicular content between cancer cells and limit the transmission of chemoresistance (Figure 3).

#### Inhibitors of Actin-Interacting Proteins

Cytochalasin D is a fungal toxin which binds to actin filament edges, preventing actin polymerization (Catalano and O’driscoll, 2020). Actin polymerization is vital in microvesiculation as well as the trafficking of MVBs toward the cell membrane; thus, cytochalasin D can inhibit EV release. It was proven that HeLa (human cervical adenocarcinoma), Panc 1 (pancreatic carcinoma), PC3, and A293 (human embryonic kidney epithelial) cells secreted exosomes carrying survivin, an anti-apoptotic protein (Khan et al., 2011). It was shown that treatment with cytochalasin D treatment decreased the release of exosomes in those cell lines, therefore reducing the concentration of survivin in the tumor environment (Khan et al., 2011). Cytochalasin D may be capable of inhibiting macropinocytosis, as macropinocytosis also involves actin-dependent pathways (Costa Verdera et al., 2017). However, its effect is not specific in targeting endocytosis mechanisms and its efficacy between different cell lines vary (Dutta and Donaldson, 2012).

#### Inhibitors Targeting Other Cytoskeleton-Related Proteins

Calpeptin is currently the most well-characterized calpain inhibitor. Calpains are cytosolic proteases and their activated forms participate in cellular activities including cell cycle progression, cell motility and cytoskeleton remodeling (Jorfi et al., 2015). Calpains are deregulated in cancer cells and thus play a role in cancer progression, apoptosis and migration. As per Figure 3, calpains promote the shedding of MVs through cytoskeletal rearrangement; thus, calpain inhibitors such as calpeptin can reduce the amount of MV shedding by cells, and they reduce cell proliferation as well. Jorfi et al. (2015) studied the chemosensitizing effect of calpeptin inhibition on the PC3 cell line, where the level of cell proliferation and apoptosis due to docetaxel treatment increased substantially in comparison to PC3 cells which did not receive calpeptin (Jorfi et al., 2015).

Chloramidine (Cl-amidine) is a calcium chelator which irreversibly binds to peptidylarginine deiminase (PAD), consequently causing post-translational protein deimination. PAD-modulated deimination of actin in the cytoskeleton is needed in order for MVs to form (Catalano and O’driscoll, 2020). Kosgodage et al. (2017) demonstrated the ability of Cl-amidine to reduce the release of exosomes as well as MVs (Kosgodage et al., 2017), showing the involvement of PAD in exosome release. Cl-amidine may inhibit EV uptake as well. It has been shown to target both CIE and CDE (Vercauteren et al., 2010) although this targeting is unspecific (Dutta and Donaldson, 2012).

Another inhibitor, NSC23766, acts on Rac1, a Rho GTPase which is involved in cytoskeletal remodeling (Catalano and O’driscoll, 2020). In cancer cells, activated Rac1 (bound to GTP) contributes to chemoresistance by contributing to angiogenesis and by binding to regulators of apoptosis (such as Bcl-2) which arouses anti-apoptotic responses from the cell (Liu et al., 2019). NSC23766 blocks the activation of Rac1 via guanine nucleotide exchange factors (GEF) Trio and Tiam1 (Catalano and O’driscoll, 2020). While NSC23766 has been shown to strongly reduce MV secretion in non-cancerous human cells (Wang et al., 2017), though it does not appear to have a significant effect when used alone in cancer cells without a second EV inhibitor (Wu et al., 2019). It may also function as an inhibitor of macropinocytosis (Hernandez et al., 2010).

Y27632 is a competitive inhibitor of ROCK1 and ROCK2, cytoskeleton-interacting members of the ROCK family (Catalano and O’driscoll, 2020). Y27632 has been used to demonstrate the relation of ROCK1 and ROCK2 to MV formation (Hartmann et al., 2015). ROCK1 and ROCK2 activate LIMK and MYLK (Li et al., 2012), which affect cofilin and myosin to stimulate cytoskeleton re-organization and the contraction of actin filaments (Kosgodage et al., 2017), both activities of which are integral to the process of microvesiculation. Y27632 competes with ATP to bind ROCK1 and ROCK2 catalytic binding sites (Catalano and O’driscoll, 2020). Y27632 acts significantly on MV-sized vesicles, reducing their secretion by 67% in PC3 cells and decreasing exosome-sized secretion by only 3% (Kosgodage et al., 2017). In a study by Li et al. (2012), tissue transglutaminase immunohistochemical staining of MDA-MB-231 (breast adenocarcinoma), U87 (glioblastoma) and HeLa cells following their treatment with Y27632 showed that MVs were not visible at their surfaces (Li et al., 2012) and the medium conditioned by Y27632-treated MDA-MB-231 and U87 cells contained a significantly lower level of MVs compared to the level prior to Y27632 treatment (Li et al., 2012).

### Inhibitors of EVs Release

**FIGURE 4:**
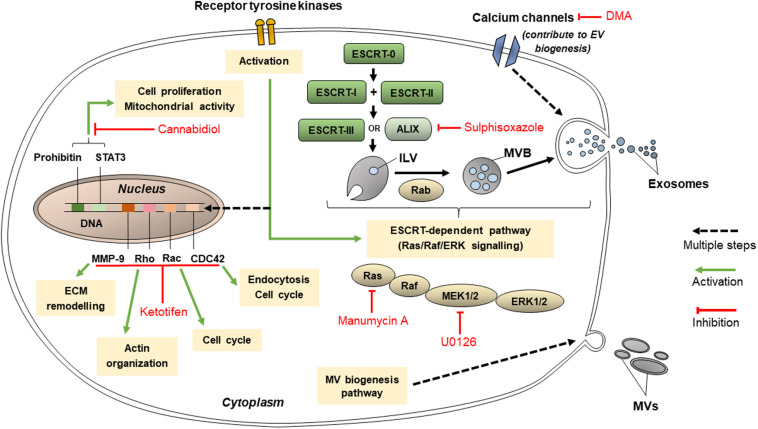
Pathways that targeted by cannabidiol, ketotifen, manumycin A, U0126, sulphisoxazole, and dimethyl amiloride (DMA) for inhibition of EV release.

Extracellular vesicles are released using many other mechanisms such as those involving protein kinases, calcium channels and other molecules, as well as the ESCRT-dependent pathway (Figure 4). These targets for inhibition of EV release may not be directly related to lipid-related pathways or cytoskeleton organization and are hence classified separately. Nonetheless, these pathways are vital in EV release, and their inhibition could decrease the conveyance of chemoresistance between cancer cells.

#### Inhibitors Targeting Protein Kinases

These may include Y27632 (described in section “Inhibitors Targeting Other Cytoskeleton-Related Proteins”), U0126, imatinib, and dasatinib. U0126 specifically and non-competitively inhibits MEK 1 and MEK 2, mitogen-activated protein kinase kinase (MAPKK) protein kinases (Catalano and O’driscoll, 2020), which prevents the activation of ERK, needed for microvesiculation to occur. U0126 is proved to reduce MV secretion (Catalano and O’driscoll, 2020) and when used in combination with gemcitabine for 72 h, increased levels of cell death in chemoresistant Suit-2 cells by 11-fold compared to the control (Becker et al., 2016). Dual treatment of another chemoresistant human pancreatic cancer cell line, MPanc-96, with U0126 and gemcitabine for 72 h also increased the levels of cell death, though not as dramatically as in Suit-2 (Becker et al., 2016). These results show that U0126 is an effective MV inhibitor able to induce chemosensitivity in chemoresistant cell lines, although its efficacy varies between cell lines.

Imatinib and dasatinib, medications for leukemia, both inhibit ATP-binding sites of the catalytic sites of bcr-abl tyrosine kinase enzymes (Catalano and O’driscoll, 2020). Phosphorylated receptor tyrosine kinases promote anti-apoptotic activity in cancer-derived exosomes (Song et al., 2016). Dasatinib was developed when myeloid leukemia cells were shown to acquire resistance to imatinib, and dasatinib was proven to be effective on all mutants of the bcr-abl tyrosine kinase enzyme (Mineo et al., 2012). Imatinib and dasatinib reduced exosome biogenesis in the chronic myeloid leukemia cell line K562 by 58 and 56%, respectively (Mineo et al., 2012).

#### Inhibitors Targeting ESCRT-Dependent Pathway of EVs Production

These include manumycin A, tipifarnib, and sulphisoxazole. Manumycin A and tipifarnib both diminish levels of exosome production primarily by targeting Ras farnesyltransferase enzymes, hence inhibiting their activation in order for Ras/Raf/ERK1/2 signaling to proceed, which is part of the ESCRT-dependent pathway of exosome biogenesis (Hanson and Cashikar, 2012; Datta et al., 2018). However, manumycin A also inhibits nSMase2 activity (Canals and Hannun, 2013) and hnRNP H1 (Datta et al., 2017). It has been suggested at low concentrations of manumycin A, nSMase2 inhibition does not occur and that both nSMase2 and Ras farnesyltransferase activity is needed in order for manumycin A to efficiently inhibit exosome biogenesis (Datta et al., 2017). Manumycin A (250 m) was found to inhibit exosome biogenesis by 50, 60, and 65% in prostate cancer cell lines C4-2B, PC3, and 22Rv1, respectively (Datta et al., 2018). Meanwhile, tipifarnib activity was found to reduce the phosphorylation of signal transducers and activators of transcription 3 (STAT3) and ERK, the latter of which is needed in exosome biogenesis, and both of which are needed for the growth of cancer cells (Datta et al., 2018). Tipifarnib (1 μM) was found to inhibit exosome biogenesis by 70% in a modified version of human prostate cancer cell line C4-2B which expressed the exosomal marker CD63 (Datta et al., 2018).

Sulphisoxazole are antibacterial drugs which, by targeting endothelin receptor type A (a G-protein coupled receptor), demonstrates inhibitory activity on components within or relating to the ESCRT-dependent pathway such as ALIX and VPS4B (which aids in ILV formation) as well as some RABs (Catalano and O’driscoll, 2020). It was proven to reduce the secretion of small EVs in breast adenocarcinoma cell lines MCF-7, MCF-10A, and MDA-MB-231 (Im et al., 2019). Most of the small EVs inhibited were likely to be exosomes, since sulphisoxazole did not inhibit the secretion of MVs from the breast cancer cell lines used (Im et al., 2019).

#### Inhibitors Targeting Other Pathways of EVs Release

Cannabidiol and SMR peptides are recently discovered to be able to inhibit EV release. Cannabidiol, a phytocannabinoid, has been discovered to inhibit EV release from HEPG2 as well as MDA-MB-231 and PC3 cell lines (Kosgodage et al., 2018). Its effect is dose-dependent and varies across different cancer cell types. It has been found to affect mitochondrial functions via reducing the expression of STAT3 and prohibitin, both of which positively regulate cell proliferation, and hence is a possible agent to sensitize chemoresistant cells to chemotherapy drugs (Kosgodage et al., 2018). Meanwhile, SMR peptides impedes mortalin expression (Huang et al., 2019). Mortalin is present in erythroleukemia K562 cell-derived EVs and has been implicated in EV release, although its precise relation is not known (Pilzer and Fishelson, 2005). SMR peptides blocked EV release in the cell lines MCF-7 and MDA-MB-231 (Huang et al., 2019). It inhibited the growth of the breast cancer cells without affecting their cell viabilities (Huang et al., 2019).

Ketotifen (an antihistamine) and dimethyl amiloride (DMA) (a derivative of amiloride, a drug to treat high blood pressure) inhibit exosome release by altering intracellular calcium levels (Khan et al., 2018; Catalano and O’driscoll, 2020). Calcium-dependent pathways are prevalent in exosome release (Khan et al., 2018). Ketotifen and DMA hinder calcium entry into cells. DMA targets calcium channels (Catalano and O’driscoll, 2020) whereas ketotifen inhibits the expression of CDC42, Rac, Rho and MMP-9 (Kim et al., 2014) as indicated in Figure 4. Ketotifen sensitized HeLa cells, as well as MCF-7 and BT549 breast cancer cells, to doxorubicin proportionally to its effect on exosome release inhibition (Khan et al., 2018). DMA was shown to decrease exosome secretion both *in vitro* and *in vivo* (Chalmin et al., 2010). An *in vitro* study using CT26 (mouse colon carcinoma), EL4 (mouse lymphoma) and H23 (human lung adenocarcinoma) cell lines showed that DMA reduced exosome release as per analysis of the culture medium (Chalmin et al., 2010). *In vivo* results of the study are discussed in section “Targeting EVs in Clinical Applications.” Additionally, as calcium channels are also involved in micropinocytosis, DMA has been shown to inhibit macropinocytosis in mammalian cells, though it has not yet been used in cancer cell lines specifically.

### Inhibitors of Endocytosis

**FIGURE 5:**
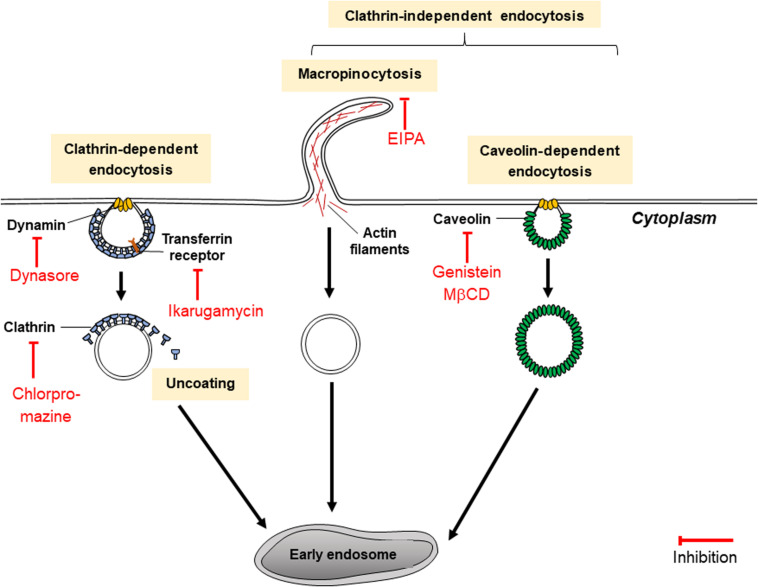
Mechanisms of endocytosis inhibitors (dynasore, chlorpromazine, ikarugamycin, genistein, and MβCD).

Pathways of clathrin-dependent and CIE may be targeted by EV inhibitors to prevent the uptake of EVs by cancer cells (Figure 5). If EV uptake inhibitors are utilized, even if EVs are released by chemoresistant cancer cells, the uptake of those EVs by chemosensitive cells will be reduced, allowing those cells to remain sensitive to chemotherapy drugs.

#### Inhibitors of CDE

Choosing the appropriate inhibitor to investigate a specific endocytic pathway can be challenging as they may be unspecific or toxic for different cell lines. Inhibitors of CDE include dynasore and ikarugamycin (IKA) as well as older inhibitors such as chlorpromazine, potassium depletion and hypertonic sucrose (Dutta and Donaldson, 2012). While potassium depletion and hypertonic sucrose are still commonly used to study CDE, they are non-specific as they may target CIE as well as CDE (Dutta and Donaldson, 2012). Potassium depletion may result in decreases of protein and DNA synthesis, whereas hypertonic sucrose results in shrinkage of cells and could affect cytoskeletal actin. Chlorpromazine blocks CDE effectively by assembling clathrin and adaptor proteins on endosomal membranes, depleting plasma membranes of clathrin (Vercauteren et al., 2010). It has also targets CIE and was discovered to be toxic to several non-cancerous cell lines (Vercauteren et al., 2010).

Dynasore and IKA are more recent inhibitors. Dynasore is a widely used CDE inhibitor which has been thoroughly characterized. But like the other CDE inhibitors, dynasore too may have non-specific effects. Dynasore non-competitively inhibits GTPase activity of the dynamin proteins dynamin1, dynamin2 and Drp1 (mitochondrial dynamin) in just seconds. Dynamin proteins are necessary for a late step of CDE which involves the production of a clathrin-coated endocytic vesicle (as indicated in Figure 5) and may participate in earlier stages of CDE (Kirchhausen et al., 2008). Dynasore inhibits the endocytosis of exosomes (Dutta and Donaldson, 2012). Dynasore may not only act as a CDE inhibitor: previous studies have shown that dynamin proteins regulate the assembly of actin filaments into bundles (Gu et al., 2010). Hence, it could be hypothesized that dynasore could have some additional effect on exosome release in cells. However, this has not been tested in cancer cells and this hypothesis is dubious since dynasore was shown to be ineffective in inhibiting exocytosis in synaptic vesicles (Newton et al., 2006). In comparison, IKA has been found to exclusively inhibit CDE in H1299, HCC366 and H1437 lung cancer cell lines (Elkin et al., 2016). It targets different receptors involved in CDE according to the cell line. In H1299 cells, it acts on transferrin receptors (TfNR) to inhibit CME (Elkin et al., 2016). 4 μM of IKA was also found to have no significant effect on cell viability of H1299 for the first 8 h; after 48 h, significant levels of apoptosis occurred and only 20% of the cells remained viable (Elkin et al., 2016). At 32 μM of IKA, the concentration of IKA which absolutely inhibits CDE, reductions in cell viability of H1299 were detected within an hour of treatment with IKA and after 8 h of incubation, only 20% of cells remained viable (Elkin et al., 2016). The cytotoxicity of IKA with other cancerous cell lines has not been thoroughly examined. The efficacy, specificity and toxicity of CDE inhibitors may be highly variable between different cell lines and suitable inhibitors must be chosen carefully for various types of research.

#### Inhibitors of CIE

Caveolin-mediated endocytosis (CavME) inhibitors include genistein and methyl-β-cyclodextrin (MβCD) as well as heparin. Genistein is a highly specific, dose-dependent inhibitor of tyrosine kinases such as the EGF receptor kinase, and pp60^*v–arc*^ and pplloBn^*gag–fes*^ kinases (Costa Verdera et al., 2017). It disrupts the arrangements of actin and prevents the mobilization of dynamin for plasma membranes, two mechanisms vital for CIE (Costa Verdera et al., 2017). Genistein has been reported to be toxic to ovarian tumor cells A2780, CaOV3, ES2, and SK-OV-3 at concentrations above 10 μM (Antosiak et al., 2017). Meanwhile, MβCD removes cholesterol from the plasma membrane, disturbing lipid rafts within it, subsequently diminishing the uptake of exosome-sized vesicles (≤150 nm) (Kosgodage et al., 2017). However, its effect is non-specific as it also inhibits exosome secretion from cells as well as other endocytic pathways such as macropinocytosis and CDE (Dutta and Donaldson, 2012). In the study by Kosgodage et al. (2017), MβCD reduced exosome secretion by 58% compared to the control. MβCD has been documented to severely impair cell morphology especially under incubation times which are longer than 2 h, using concentrations of MβCD higher than 5 mM, which is in the range of concentrations of MβCD typically used to inhibit exocytosis (Kosgodage et al., 2017). MβCD is typically used in combination with lovastatin, an inhibitor of cholesterol synthesis, to prevent cholesterol being synthesized in cells and replacing the cholesterol in the plasma membrane extracted by MβCD (Kosgodage et al., 2017).

Heparin, an anticoagulant, competitively inhibits cancer cell surface receptors which depend on heparin sulfate proteoglycan (HSPG) coreceptors for the uptake of exosomes when HSPGs are present (Sento et al., 2016). An example of a HSPG which is necessary for exosome biogenesis is Syndecan (Sento et al., 2016). Heparin primarily acts on the receptors of treated cells rather than the receptors on treated exosomes, as shown by a study using the urothelial carcinoma cell line SW780 (Franzen et al., 2014). In another study by Christianson et al. (2013), it was observed that exosome uptake in U87 cells was reduced by 55% compared to the untreated control. In addition, exosome uptake was reduced significantly in medium depleted of Ca^2+^ compared to medium consisting Ca^2+^ (Christianson et al., 2013). It was concluded that heparin inhibits exosome internalization in a dose- and charge density-dependent way (Christianson et al., 2013).

5-(N-ethyl-N-isopropyl)amirolide (EIPA) is one chemical derivative of amiloride which has been regularly used to inhibit macropinocytosis. EIPA lowers sub-membranous pH by targeting Na^+^/H^+^ exchangers (Koivusalo et al., 2010), and like NSC23766, disrupts Rac1 activation as well as the assembly of actin, hence reducing the uptake of EVs as demonstrated using HeLa cells (Costa Verdera et al., 2017). While EIPA is regarded as effective as a pharological inhibitor of macropinocytosis, it inflicts “collateral damage” on treated cells with effects on ion transport, intracellular pH and the cytoskeleton (Costa Verdera et al., 2017). Imipramine has been suggested as an alternative; intracellular pH of treated cells has been proven to recover following treatment with imipramine, and imipramine was shown to effectively reduce macropinocytosis activity in a range of cell lines including mammary carcinoma 4T1 cells (Lin et al., 2018).

### Difficulties, Limitations, and Considerations in EVs Inhibition

The challenges in using EV-inhibiting drugs to tackle chemoresistance relate to the efficacy, specificity and toxicity of inhibitors; lack of knowledge regarding the precise mechanism of several inhibitors; and adapting functional and potent inhibitor drugs for clinical use. These challenges are further complicated by still-evolving knowledge regarding EV-related pathways.

First, many EV inhibitors vary in their efficacy (between different cell lines and cell types) and their specificity. In a clinical setting, this could lead to difficulty in choosing an appropriately efficacious inhibitor for treatment. Some EV inhibitors may be so specific as to target a single protein involved in EV biogenesis or uptake such as indomethacin which targets ABCA3; but some may have a wide range of targets with varying downstream effects, such as manumycin A which may target Ras farnesyltransferase and nSMase2 enzymes. Some EV inhibitors may target both exosomes and MVs (such as cannabidiol, Cl-amidine, and imipramine), whereas others (such as calpeptin, cytochalasin D, and Y27632) may target the exocytosis of only one subtype of EV. Some EV inhibitors may even target pathways of EV release as well as those of EV uptake, since those pathways may share similar mechanisms, for instance cytochalasin D, MβCD and simvastatin. A particular inhibitor may inhibit the release of certain EV populations but promote the release of others. For example, GW4869 inhibits exosome release but was reported to stimulate MV release in breast adenocarcinoma SKBR3 cells (Menck et al., 2017).

Many EV inhibitors do not completely inhibit EV release or uptake when used independently. This is usually because the activity of each EV-related pathway contributes only a proportion of total EV secretion or release, and most inhibitor drugs do not inhibit all the pathways of EV release or EV uptake with equal efficacy. All EVs secreted by a cell may have been the result of various EV release pathways, and all EVs taken up by a cell may have been ingested by any endocytosis or macropinocytosis pathway. For example, a study by Costa Verdera et al. (2017) reported that genistein and EIPA, inhibitors of CIE and MP, respectively, individually inhibited total EV uptake in A431 cells and HeLa cells by only about 50%; when genistein and EIPA were used in combination, EV uptake was almost totally suppressed, which suggested that both CIE and MP are major contributors to EV uptake (Costa Verdera et al., 2017). Similarly, in a study by Datta et al. (2017), exosome production reduction levels in prostate cancer cell lines (PC3 and 22Rv1) ranged from 50 to 60% using 250 nm of manumycin A, but those levels rose significantly when 10 μM of GW4869 was used together (Datta et al., 2017). The efficacy of using both GW4869 and manumycin A, nSMase2 (or ESCRT-independent) and ESCRT-dependent inhibitors respectively, in the study proved that both ESCRT-independent and ESCRT-dependent activities are vital in exosome biogenesis in prostate cancer cell lines. Therefore, in order to completely inhibit a subpopulation of EVs, at least two inhibitors should be used.

In clinical settings, the prospect of using two EV inhibitors together with chemotherapy is bound to raise questions regarding managing the side effects of this treatment approach. The relative cytotoxicity of different EV inhibitors to different cell lines may also raise hurdles in research as cytotoxic effects may distort experimental results. EV inhibitors may be toxic at the same concentration found to be optimum for EV inhibition, especially after prolonged incubation. One example of such an inhibitor is IKA, which already reduces cell viability within an hour of treatment and impairs the morphologies of cells and cell organelles under prolonged incubation (Elkin et al., 2016). Another toxic effect of EV inhibitors may include the inhibition of EVs essential to regulate healthy physiological processes in cells unrelated to chemoresistance (Maacha et al., 2019). Since EV inhibitors used should not introduce additional effects unrelated to the pathway under study, the cytotoxic effects of some inhibitors pose some challenge to research. In addition, it has been noted by Dos Santos et al. (2011) that, regardless of their toxicity, any endocytosis inhibitors chosen (e.g., chlorpromazine and genistein) should not cause reorganization of the cytoskeleton as this may affect EV uptake mechanisms unrelated to actin, thus distorting the functions of plasma membrane proteins (Santos et al., 2018). This may obfuscate experimental results. As a treatment approach, usage of EV inhibitors which are toxic to normal cells may eventually lead to severe damage to organs.

While there may not be a perfect inhibitor yet, the pursuit to discover fast-acting inhibitors which have specific, reversible and minimally toxic effects should continue (Dutta and Donaldson, 2012). For the study of endocytic and exocytic mechanisms related to chemoresistance in cancer cells, using EV inhibitors alone may not be sufficient, since EV inhibitors may have low specificities. A possible complement to this research for the confirmation of results is the knockdown of proteins and regulators (Costa Verdera et al., 2017) associated with EV-related pathways such as ALIX, Vsp4 and Rab proteins for exosome biogenesis; aSMase, cholesterol, ARF6 and ARF1 for microvesiculation; dynamin-1, dynamin-2, and adaptor protein-2 (a major coat protein) for CDE (Mettlen et al., 2018); ARF6, CDC42, RhoA and caveolin-1 for CIE; and Rac1 for macropinocytosis.

## Targeting EVs in Clinical Applications

At this stage, most drug inhibitors of EV release or uptake have only been tested in the context of chemoresistance under *in vitro* conditions using various cancer cell lines. However, DMA was assessed in a pre-clinical study by Chalmin et al. (2010) to determine their ability to curb exosome release *in vivo*. *In vivo*, DMA reduced exosome release into the bloodstream of mice carrying tumors (Chalmin et al., 2010). Combination therapy of tumor-carrying mice with DMA and the chemotherapy drug cyclophosphamide staunched tumor growth by 50% or more compared to the untreated controls (Chalmin et al., 2010). These results were consistent among the three mouse-tumor models created using two mouse cancer cell lines, EL4 and TS/A, and one human cancer cell line, CT26. Reduced tumor growth was attributed to DMA sensitizing the tumor cells to cyclophosphamide. As such, DMA holds potential in improving the efficacy of chemotherapy treatment. More pre-clinical research involving EV inhibitors discussed in this review is required prior to establishing the ability of EV inhibitors to mitigate chemoresistance in a clinical context.

Alternatively, EVs may be targeted using a genetic approach. For example, knockdown of the nSMase2 gene suppressed EV release and inhibited angiogenesis and metastasis in a xenograft mouse model of breast cancer (Kosaka et al., 2013). However, nSMase2 is essential to intercellular communication in all cells, including healthy cells. Like EV inhibition, in clinical settings, the knockdown of nSMase2 may also adversely affect the physiological activities of non-cancerous cells. In addition, nSMase2 knockdown failed to impact EV synthesis and release in the prostate cancer cell line PC3 (Phuyal et al., 2014). Prior to clinical implementation of this approach, other genes related to EV production in specific cancer types should be analyzed as potential targets. Bobrie et al. (2012) also utilized short hairpin RNA (shRNA) specific to the Rab27a gene to silence its expression, after establishing that Rab27a is involved in exosome secretion (Bobrie et al., 2012). The results of Rab27a inhibition varied between the two breast cancer cell lines used, 4T1 and TS/A. Using a mouse model, it was shown that shRNA targeting Rab27a decreased growth and metastasis of 4T1 whereas the same targeting did not affect growth or metastasis of TS/A. The study by Bobrie et al. (2012) highlights the importance of specific treatments for different types of cancer cells; even two cell lines derived from the same tissue can respond very differently to a treatment.

Rather than targeting EV biogenesis or uptake, EVs may also be removed from the circulation. Marleau et al. (2012) strategized a hemofiltration system targeting breast cancer-derived EVs which expressed human epidermal growth factor receptor type 2 (HER2). EVs expressing HER2 were found to stimulate tumor growth and induce the formation of premetastatic niches, promoting metastasis (Marleau et al., 2012). HER2-expressing EVs promote chemoresistance and reduce the effectiveness of breast cancer treatments (Marleau et al., 2012) so elimination of these EVs may prevent metastatic formations. This treatment strategy holds promise, but it is specific to breast cancer. For other types of cancers, different receptors expressed by EVs secreted by specific kinds of cancers should be investigated as potential targets in the hemofiltration system strategy.

In another study, antibodies to CD9 and CD63, glycoprotein receptors present on the surface of EVs, were used to deplete EVs in a xenograft mouse model of breast cancer (Nishida-Aoki et al., 2017). The study yielded a pronounced reduction in metastasis, especially for treatment using anti-CD63 antibodies. However, unlike the hemofiltration system described by Marleau et al. (2012), this procedure does not specifically target only cancer-derived EVs. EVs secreted by normal body cells will also be targeted, preventing intercellular communication between healthy cells and adversely affecting regular cellular activities within the body. This treatment also did not impact the growth of the primary tumor site. In the future, the effect of combined treatments of anti-CD63 and chemotherapy drugs on animal breast cancer models could be investigated to reduce primary tumor growth while decreasing metastatic activity. In addition, antibodies targeting receptors more specific to breast cancer (and other types of cancers) can be used instead to specifically target cancer-derived EVs. This will reduce the side effects of this treatment strategy in clinical settings.

Extracellular vesicle inhibition, control of EV-related gene expression and hemofiltration of EVs all prevent or reduce intercellular communication between cancer cells. When these methods are combined with chemotherapy, they can be expected to result in the repression of EV-mediated mechanisms of chemoresistance such as EMT (Figure 1). Chemoresistance is linked to metastasis and tumor relapse (D’alterio et al., 2020), two main causes of deaths among cancer patients (Avanzini and Antal, 2019; Dillekås et al., 2019). Thus, suppressing EV-mediated chemoresistance can be speculated to reduce rates of metastasis and tumor relapse among cancer patients receiving chemotherapy.

Clinical progresses in targeting EV-mediated chemoresistance are slowed by gaps in knowledge regarding the roles of EVs in mechanisms of chemoresistance. Considering this, investigations into existing drugs which inhibit the release or uptake of EVs may yield discoveries regarding their suitability for use in cancer treatments. Their suitability for use may be defined in terms of their efficacy and toxicity when used alongside chemotherapy drugs. Further research into novel methods to target EV-mediated intercellular communication between cancer cells may aid in planning anticancer therapies while minimizing side effects.

## Conclusion

**FIGURE 6:**
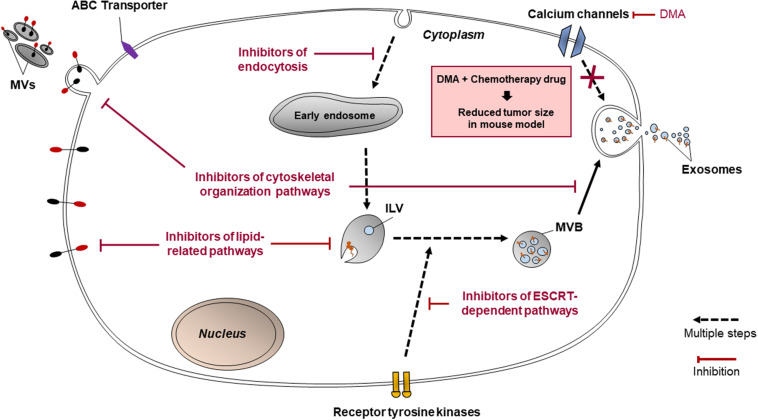
Summary of possible targets for the inhibition of EV biogenesis, release, and uptake.

Exosomes and MVs contribute significantly to chemoresistance in many cancer cell lines via mechanisms such as the transfer of miRNAs, which activate anti-apoptotic signaling and DDR, and by enhancing other processes such as chemotherapy drug efflux, immunosuppression, alteration of cytosolic pH and EMT. Hence, the inhibition of EV release and uptake could be a promising companion treatment to enhance the efficacy of chemotherapy drugs. Since both exosomes and MVs can be released and taken up by cancer cells via multiple pathways, and since those pathways involve diverse and specific mechanisms, there are many possible targets for the inhibition of EV release and uptake. Such targets may include proteins, transporters, protein kinases and enzymes involved in lipid-related pathways of EV release, cytoskeletal organization, the ESCRT-dependent pathway of EV release, clathrin-dependent and clathrin-independent endocytosis (summarized in Figure 6).

Extracellular vesicle inhibitors may have the capacity to sensitize chemoresistant cells under both *in vivo* and *in vitro* conditions; in the case of DMA, this has already been proven using a mouse model. Yet, this strategy requires much investigation as preclinical studies of chemoresistance using EV inhibitors are limited. Pathways related to EV biogenesis, release and uptake mechanisms have yet to be thoroughly explained, and the mechanisms of existing EV inhibitors must be studied further, especially for the use of inhibitors in combination to fully block all avenues of EV production or uptake. More efficacious EV-inhibiting drugs must be developed before they can be implemented in a clinical setting. Further investigation into EV release and uptake pathways is required in order to fully describe the mechanisms involved, and to develop more advanced EV inhibitors capable of targeting those specific mechanisms to counteract chemoresistance. This is vital in advancing the effectiveness of anticancer treatments.

## Author Contributions

RH performed searching, analyzed data, and wrote the manuscript. NK, B-HG, and W-LL contributed to the conceptual idea, reviewed the drafts, supervised the writing process, and provided important information for the completion of this manuscript. LM and ZF reviewed the drafts and refined the manuscript. All the authors revised and approved the final version of the manuscript.

## Conflict of Interest

The authors declare that the research was conducted in the absence of any commercial or financial relationships that could be construed as a potential conflict of interest.
